# Advances in Management of Neuropsychiatric Syndromes in Neurodegenerative Diseases

**DOI:** 10.1007/s11920-019-1058-4

**Published:** 2019-08-08

**Authors:** Jeffrey Cummings, Aaron Ritter, Kasia Rothenberg

**Affiliations:** 1 Department of Brain Health, School of Integrated Health Sciences, University of Nevada Las Vegas (UNLV), 4505 S Maryland Pkwy, Las Vegas, NV 89154 USA; 2 Cleveland Clinic Lou Ruvo Center for Brain Health, 888 W Bonneville Ave., Las Vegas, NV 89106 USA; 3 Cleveland Clinic Lou Ruvo Center for Brain Health, Cleveland, OH USA

**Keywords:** Neurodegenerative disorders, Neuropsychiatric syndromes, Alzheimer’s disease, Parkinson’s disease, Depression, Psychosis, Apathy, Agitation

## Abstract

**Purpose of Review:**

Neuropsychiatric syndromes (NPS) are common in neurodegenerative disorders (NDD). This review describes the role of NPS in the diagnosis of NDD, criteria for the diagnosis of NPS, management of NPS, and agents in clinical trials for NPS.

**Recent Findings:**

NPS play an increasingly important role in the diagnosis of NDD. Consensus diagnostic criteria have evolved for psychosis, depression, agitation, and apathy in NDD. With one exception—pimavanserin is approved for the treatment of hallucinations and delusions in Parkinson’s disease—there are no drugs approved by the FDA for treatment of NPS in NDD. Trials show that atypical antipsychotics reduce psychosis in AD and in Parkinson’s disease, although side effect concerns have constrained their use. Antidepressants show benefit in treatment of Parkinson’s disease with depression. Several agents are in clinical trials for treatment of NPS in NDD.

**Summary:**

Neuropsychiatric syndromes play a major role in NDD diagnosis. Clinical criteria allow recognition of NPS in NDD. Psychotropic medications are often useful in the treatment of NPS in NDD; efficacious, safe, and approved agents are needed.

## Introduction

Neuropsychiatric syndromes (NPS) are common in neurodegenerative disorders (NDD). They occur in nearly all patients with Alzheimer’s disease (AD) [1–3]. NPS have many adverse consequences including distress for the patient, reduction of patient and caregiver quality of life, increased risk of institutionalization, and increased cost [4–7].Depression and psychosis are associated with more rapid cognitive decline in AD [8••]. NPS are present in the prodromal phase of AD and other NDD and increase in frequency through the course of the illnesses [3, 9–11].

Despite their high prevalence and serious consequences, the only agent approved by the Food and Drug Administration (FDA) for any NPS of any NDD is pimavanserin for treatment of hallucinations and delusions of Parkinson’s disease (PD) psychosis. Past trials of antipsychotics suggest efficacy in psychosis and agitation; interest in advancing development of these agents for NPS in NDD may be limited by loss of patent protection and the generic status of many of these drugs. In the recent past, progress has been made toward developing effective therapies for NPS. Here we review advances in the pharmacologic treatment of the NPS of AD and related dementias. We discuss the role of NPS in diagnosing NDD, the definition of NPS, current approaches to treating NPS in NDD, and clinical trials and drug development for psychotropic agents used for NPS in NDD. We emphasize novel mechanisms and innovative approaches to trials and pharmacotherapy.

## Role of Neuropsychiatric Syndromes in Diagnosing Neurodegenerative Disorders

Neuropsychiatric syndromes play an increasingly important role in the diagnosis of NDD. This reflects the growing recognition of the importance of NPS as expressions of neurological disease and the unique association between NPS and specific NDD. Diagnosis of probable behavioral variant frontotemporal dementia (bvFTD) requires the presence of NPS. Other diagnostic criteria allow NPS to fulfill profiles of diagnostic criteria but do not specifically require the presence of NPS. These include the National Institute of Aging-Alzheimer’s Association (NIA-AA) definition of dementia, the NIA-AA definition of AD-type dementia (AD), the criteria for Dementia with Lewy bodies (DLB), the definition of vascular cognitive impairment (VCI), and criteria for progressive supranuclear palsy (PSP) and corticobasal degeneration (CBD). NPS are supportive of the diagnosis of PD dementia.

In the criteria for bvFTD, if the neuropsychological profile for bvFTD is met, then the patient must have at least two of the following behavioral symptoms: early behavioral disinhibition; early apathy or inertia; early loss of sympathy or empathy; early perseverative, stereotyped or compulsive/ritualistic behavior; or hyperorality and dietary changes [12]. If the neuropsychological profile is not met, then the patient must have three of the categories of behavioral changes. For bvFTD, behavioral changes represent the core changes required for the diagnosis.

The NIA-AA approach to AD begins by identifying the syndrome of all-cause dementia. This includes cognitive or behavioral symptoms that interfere with work or activities, represent a decline from a previous level of function, are not better explained by delirium or a psychiatric disorder, are well-documented by neuropsychological assessments, and have at least two of the following features: memory impairment; impaired reasoning and complex thinking; impaired visual spatial abilities; impaired language; or changes in personality, behavior, or comportment [13]. This approach to dementia allows “changes in personality, behavior, or comportment” to be one of two clinical features leading to a diagnosis of dementia. In a second step, the NIA-AA criteria define AD dementia by the presence of a dementia syndrome as previously defined plus insidious onset, and gradual worsening. It may have either an amnestic or a non-amnestic presentation. Building on the criteria for dementia allows for behavior to be one of two necessary elements for a diagnosis of AD dementia [13•].

A similar strategy for diagnosis of dementia and AD is adopted by the Diagnostic and Statistical Manual, 5th ed. [14]. This approach defines major neurocognitive disorder as having evidence of significant decline from past cognitive ability, impairment of cognitive performance sufficient to interfere with activities of daily living (ADL), and not exclusively explained by a delirium or some other mental disorder. The clinical symptoms include memory impairment, language abnormalities, visuospatial disturbance, executive dysfunction, attention impairment, and social cognition decline [15]. The inclusion of social cognition as a key feature allows major cognitive disorder to be identified by decline in empathy, sympathy, social judgment, responsiveness to social cues, and appropriate social behavior. Major neurocognitive disorder due to AD requires the presence of memory impairment plus impairment of at least one of the other domains. In this setting, compromised social cognition in addition to memory impairment would allow fulfillment of the clinical criteria for major neurocognitive disorder due to AD.

There are four core clinical features of DLB, two of which must be present to allow a diagnosis of DLB in a patient with progressive cognitive decline [16••]. These core features include fluctuating cognition, recurrent visual hallucinations, rapid eye movement (REM) sleep behavior disorder, and parkinsonism. One can make a diagnosis with no behavioral features based on fluctuating cognition and parkinsonism, but behavioral features including hallucinations and REM sleep behavior disorder are core features that can fulfill the criteria for probable DLB and are invoked in a majority of DLB diagnoses [16••].

Diagnostic criteria for PD dementia [17] identify behavioral symptoms (apathy, depressed or anxious mood, hallucinations, delusions, excessive daytime sleepiness) as supportive of the diagnosis but note that the absence of behavioral symptoms does not exclude the diagnosis.

The diagnostic criteria for corticobasal degeneration include insidious onset and gradual progression in an individual 50 years of age or older. Symptoms must be present for a minimum of 1 year and include the corticobasal syndrome or a frontobehavioral syndrome plus at least one corticobasal feature, such as asymmetric limb rigidity or bradykinesia, asymmetric limb dystonia, asymmetric limb myoclonus, orobuccal or limb apraxia, cortical sensory deficit, or alien limb phenomenon [18•]. Thus, a behavioral type of frontobehavioral syndrome plus one corticobasal syndrome feature allows the diagnosis of corticobasal degeneration.

Diagnostic criteria for PSP do not allow behavioral abnormalities to meet criteria for level 1 certainty but do allow including behavioral abnormalities (frontal cognitive/behavioral presentation) to achieve a level 2 diagnostic certainty. There are three levels of certainty in this diagnostic approach [19•].

Other sets of diagnostic criteria for cognitive disorders also include reference to behavioral features. Vascular cognitive impairment (VCI) requires onset of the clinical syndrome to be related to a cerebrovascular event and evidence of decline in frontal executive functioning, plus one of the following: gait disturbance, urinary symptoms, or personality and mood changes. There must also be evidence on computerized tomography (CT) or magnetic resonance imaging (MRI) of cerebrovascular disease. In this set of criteria, personality and mood changes can contribute to meeting the criteria for diagnosis of VCI [20•].

Only a few diagnostic criteria sets do not include an overt inclusion of a behavioral alternative to meet diagnostic criteria. The NIA-AA criteria for mild cognitive impairment (MCI) due to AD do not include reference to behavioral changes [21•]. Similarly, primary progressive aphasia, including progressive non-fluent aphasia and progressive semantic dementia, does not include specific reference to behavioral features that would allow meeting the diagnostic criteria [22•].

Review of these criteria demonstrates that behavioral features are commonly recognized in NDD and contribute importantly to diagnostic approaches. Progress in understanding the biology of NPS will likely lead to an enhanced ability to use them for classification, course prediction, treatment, and therapeutic monitoring of NDD.

## Diagnostic Criteria for Neuropsychiatric Syndromes

Neuropsychiatric syndromes are to be distinguished from symptoms. Symptoms are subjective phenomena, experienced by the individual. Syndromes are constellations of clinical features, signs, symptoms, or phenomena that are recognized by a clinician and comprise a medical condition. Most clinical phenotypes discussed here are syndromes.

Specific diagnostic criteria have been developed for psychosis in AD [23•], depression in AD [24•], apathy in AD and other NDD [25•], and agitation in cognitive disorders including AD [26••]. Criteria for specific neuropsychiatric syndromes in PD include psychosis in PD [27•] and depression in PD [28•].

Consensus criteria are typically derived by panels of experts. The sensitivity, specificity, and reliability of the criteria are shown following the introduction of the criteria and they are often refined based on clinical experience. For this reason, many criteria are labeled “provisional” or “draft” when they are first published. Criteria are critical to allow a common vocabulary for diagnosis. They facilitate many types of research including epidemiology, studies of pathophysiology, relationships to burden, and financial cost.

Criteria for neuropsychiatric syndromes are critical to facilitate clinical trials. In drug development programs, diagnostic criteria for neuropsychiatric syndromes allow the identification and quantification of the unmet need. They characterize the eventual “indication” that will comprise the reason for prescribing the drug. They are critical to planning clinical trials and anticipating the challenges of recruitment. They are essential when presenting the trial results to the FDA where they allow regulatory officials to understand who is included in the trial and what specific outcomes can be anticipated. Diagnostic criteria are essential to inform prescribers once a drug is approved and to explain the use of the medication to patients, caregivers, insurers, pharmacy benefit managers, regulatory oversight agencies, and advocacy groups.

## Current Approaches to Treating Neuropsychiatric Syndromes in Neurodegenerative Disorders

The usual clinical approach to treating neuropsychiatric syndromes in NDD is to extrapolate from the use of psychotropic agents used in idiopathic psychiatric disorders for similar phenomena. Approved antidepressants are used for the treatment of depression in NDD; antipsychotics are used for the treatment of psychosis (hallucinations and delusions) and agitation in NDD; mood stabilizers are used in the management of agitation in NDD; anxiolytics are applied to anxiety in NDD; stimulants are prescribed for apathy in NDD; and hypnotics are used for sleep dysregulation in NDD. This approach is based on the approval of antidepressants for the indication of major depressive disorder, antipsychotics for schizophrenia or bipolar illness, mood stabilizers for bipolar disorder, stimulants for attention deficit disorder, anxiolytics for generalized anxiety disorder, and hypnotics for insomnia. This extrapolation is based on the clinical similarity of symptoms and behaviors of the neuropsychiatric syndromes occurring in NDD and non-NDD circumstances and the absence of alternatives. The implementation of treatment by analogy has been called the “the therapeutic metaphor” for the use of psychotropic agents [29]. However, patients with NDD are excluded from trials of patients used for testing of antidepressants, antipsychotics, anxiolytics, stimulants or hypnotics, and no data concerning the use of psychotropic medications in patients with NDD are available at the conclusion of trials involving patients with conventional psychiatric disorders. The extension of these therapies from non-NDD to NDD patients is based on many untested assumptions that may result in treating patients with agents that are ineffective or have safety and tolerability issues that differ from those observed in clinical trials of patients without NDD. The change of the clinical phenotype by the features of the NDD (e.g., apathy, cognitive impairment, sleep disturbances) and the brain changes induced by the NDD may alter the efficacy or side effect profile of the psychotropic agent. Likewise, brain changes may alter the necessary doses to impact behavior. Most patients with NDD are elderly compared to those with idiopathic disorders, and this may affect the pharmacokinetics and the pharmacodynamics of an agent. Doses of psychotropics used in the elderly are often lower than doses used in idiopathic disorders occurring in younger individuals; for example, the doses of antipsychotics that appear to be efficacious and tolerated in older AD patients with agitation are much lower than those used for psychosis in younger patients with schizophrenia. Data generated on use of psychotropic agents within patients with the NDD are vital for guiding use of psychotropics in these conditions.

## Treatment of NPS in NDD

Only one agent—pimavanserin—is currently approved by the US FDA for the treatment of a neuropsychiatric syndrome of a NDD. This agent is approved for the treatment of hallucinations and delusions occurring in PD psychosis [30••]. All other use of psychotropics is “off label,” although it may be necessary for best practices, based on extensive experience, and endorsed by treatment guidelines [31, 32]. Table 1 summarizes current recommendations for treatment of NPS in NDD.

**Table 1:** Recommended treatments for neuropsychiatric syndromes

Neuropsychiatric syndrome	1st-line therapies*	2nd-line therapies	3rd-line therapies
Agitation in AD	Citalopram (10–30 mg/day)** Risperidone (0.5–1 mg/day)	Aripiprazole (10 mg/day) Carbamazepine (300 mg/day) Dextromethorphan/quinidine (20/10 mg BID) Olanzapine (5–10 mg/day) Quetiapine (200 mg/day) Trazodone (50–100 mg/day)	Lamotrigine (25–100 mg/day) THC (2.5–7 mg/day)
Apathy in AD	Methylphenidate (20 mg/day)	Modafinil (200 mg/day)	
Depression in AD	Citalopram (10–40 mg/day)** Escitalopram (5–20 mg) Sertraline (50–150 mg)	Aripiprazole as augmentation (2 mg–15 mg/day) Bupropion (100 mg–300 mg/day) Carbamazepine (augmentation) (300 mg/day) Duloxetine (20–60 mg/day) Fluoxetine (20–40 mg/day) Mirtazapine (7.5–30 mg/day) Paroxetine (10–40 mg/day) Quetiapine as augmentation (25–200 mg/day) Venlafaxine (37.5–225 mg/day)	Electroconvulsive therapy Tricyclic antidepressants
Depression in PD	Pramipexole (0.3–4.2 mg/day) Ropinirole (10 mg/day)	Citalopram (10–20 mg/day) Desipramine (25–75 mg/day)*** Nortriptyline (25–75 mg/day)*** Sertraline(25–50 mg/day)	Electroconvulsive therapy Buproprion (100–300 mg/day) Duloxetine (30–60 mg/day) Mirtazapine(30 mg/day) Paroxetine (10–40 mg/day) Venlafaxine (37.5–225 mg/day)
Psychosis in PD	Pimavanserin (40 mg/day)	Clozapine (6.25–50 mg/day) Quetiapine (25–100 mg/day)	Risperidone (0.5–2 mg/day) Olanzapine (5–7.5 mg/day)

*Initiation of pharmacological interventions should occur after non-pharmacological approaches, cognitive enhancers, and comprehensive assessment of medical and environmental factors has been completed

**Maximum recommended dose for citalopram in patients over the age of 60 is 20 mg/day

***TCA should not be used in patients with cognitive impairment

### Overview

Psychotropics are commonly used in AD and other NDD despite the absence of a specific FDA-approved indication in this setting. It is incumbent on the clinician in these circumstances to weigh potential benefit and harm to the patient, consider the urgency and the magnitude of the threat, review the consequences of not treating, and assess the appropriateness of non-pharmacological interventions prior to initiating use of a psychotropic agent [32, 33]. A shared decision-making paradigm to include family caregivers or others is an important aspect of the decision to treat [34].

### Treatment of Agitation in AD

An unanswered question concerning agitation in NDD is whether there are different types of agitation and if these differing phenoptypes may respond differentially to treatments used for agitation. For example, among patients with agitation treated with citalopram, those with the mild-moderate symptoms appeared to respond best while those with more severe agitation demonstrated less efficacy and more side effects [35]. Different brain circuits may be involved in different types of behavioral dysregulation and may respond differentially to antipsychotics, antidepressants, or mood stabilizers [36]. This question represents an area where further study is warranted.

The potential benefit of antipsychotics must be weighed against the significant risks to patients, such as cerebrovascular adverse events and mortality. Antipsychotic use may have less risk of premature death or need for medical care in cases where careful control for cardiovascular risk factors was implemented [37]. Atypical antipsychotics are more beneficial than placebo and are associated with decreased caregiver burden, but the potential for adverse effects limits their overall usefulness [38, 39]. Not all atypical antipsychotics have been studied in the context of NPS of NDD, and the class effects are not established.

There is limited evidence to support the use of typical antipsychotics to manage aggression and agitation in clinically acute settings. Efficacy of the agents is modest [40]. Haloperidol is useful in treatment of aggression with agitation (but not general agitation behaviors, such as wandering or verbal agitation) [41]. Typical antipsychotics are not recommended in non-emergent treatment of agitation in dementia [42]. The use of typical antipsychotics in NDD even in acute situations has risk.

Atypical antipsychotics have a lower risk of certain side effect including parkinsonism, acute dystonic reactions, and tardive dyskinesia. Atypicals including risperidone, olanzapine, and aripiprazole are alternatives for use in management of severe agitation, aggression, and psychosis associated with AD where there is risk of harm to the patient and/or others [43–46]. Risperidone has the best evidence for short-term efficacy (6–12 weeks) in patients with agitation [38]. A meta-analysis of four large placebo-controlled clinical trials supported risperidone’s efficacy in the management of agitation and aggression even in severely impaired AD patients [47]. In AD patients with psychosis or agitation who had responded to risperidone therapy for 4–8 months, discontinuation of risperidone was associated with an increased risk of relapse [48•]. Risperidone may be considered as an option for short-term intervention in cases of acute, treatment-resistant agitation in AD and may be useful in long-term management of agitation where the agitation syndrome has become chronic.

Studies with quetiapine have provided mixed results. Ballard et al. [49] found no treatment benefit in reducing agitation compared to placebo. Zhong et al. [50] showed improvement of agitation in patients given 200 mg of quetiapine daily.

Olanzapine has been used for treatment of psychosis in AD [45]. It has anticholinergic effects and may decrease cognition while increasing the risk of anticholinergic delirium symptoms [51]. Olanzapine use in the treatment of delusions and hallucinations in PD has resulted in exacerbation of parkinsonism [52].

Brexpiprazole, a novel compound structurally similar to aripiprazole (the mechanism of action includes reduced partial agonism for D2, 5HT1A receptors, and enhanced antagonism for 5-HT2A and α1-adrenoreceptors), is being tested for agitation associated with AD [53–55].

Data supporting use of anticonvulsants/mood stabilizers are strongest for carbamazepine [56]. Its use is limited by the risk for side effects such as dizziness, sedation, ataxia, and confusion and the more rare but significant adverse effects of inappropriate antidiuretic hormone with hyponatremia, cardiac, and hepatotoxicity [57]. Patients should be informed of the black box warnings for aplastic anemia, agranulocytosis, and rare but sometimes fatal dermatologic adverse reactions.

The evidence for valproate as a treatment for agitation is mixed, with a meta-analysis of pooled results concluding that valproate is ineffective in reducing agitation in dementia and is associated with unacceptable rates of adverse events, notably sedation [43, 58]. There is a risk that valproate may worsen agitation compared to placebo as measured by both the Neuropsychiatric Inventory (NPI) and Cohen-Mansfield Agitation Inventory (CMAI) [59]. Among other treatments from this category, topiramate showed limited efficacy [60]. Gabapentin, lamotrigine, oxycarbamazepine, and levetiracetam have been the subject of observational or uncontrolled studies and are worthy of further investigation. One study suggested that lamotrigine may be effective and may make it possible to avoid increasing the dosage of antipsychotic medications prescribed to elderly patients with cognitive impairment [61].

Trazodone, a hypnotic and antidepressant (pharmacologically, a serotonin antagonist and reuptake inhibitor), is used for management of insomnia and night time behavioral disturbances, irritability, agitation, and aggression in AD. Trazodone has a favorable safety profile if administered in small doses and appears to produce a stabilization of the circadian rhythms in individuals with AD [62••]. Because of its hypnotic properties, trazodone may be particularly useful in patients with nocturnal agitation.

There is growing interest in selective serotonin reuptake inhibitors (SSRIs) to target agitation and aggression in dementia. A recent large randomized controlled trial found that citalopram significantly reduced agitation and caregiver distress compared to placebo. Worsening cognition and QT prolongation were significantly more common in the citalopram group (30 mg/day) [63]. Patients in the study were required to have treatment-requiring levels of agitation; those with major depression or psychosis requiring antipsychotics were excluded. Assessment of the 20 mg (the maximum dose recommended by the FDA in adults over 60) has not been conducted. Comparator studies indicate sertraline and citalopram are probably as effective as risperidone in treating agitation in dementia, especially among those with mild-moderate agitation severity [64].

Dextromethorphan/quinidine, a combination drug containing dextromethorphan, an n-methyl-d-aspartate receptor antagonist and high affinity sigma-1 receptor agonist, and the antiarrhythmic agent, quinidine, is the first FDA-approved drug for the treatment of pseudobulbar affect [65]. A recent study in patients with AD demonstrated that dextromethorphan/quinidine significantly improved AD-associated agitation, reduced caregiver burden, and was generally well tolerated [66••]. Two phase 3 studies are in progress.

Tetrahydrocannabinol (THC) studies have provided conflicting evidence regarding reduction of agitation. Low-dose THC (4.5 mg daily) did not significantly reduce NPS after 3 weeks, though it was well tolerated [67]. Previous studies with THC (2.5–7 mg daily) reported positive effects on NPS in dementia [68, 69].

### Treatment of Depression in AD

Studies of depression in AD have usually used the Diagnostic and Statistical Manual definition of major depression and research definitions of AD based on clinical criteria. Clinically defined populations are heterogeneous with regard to the underlying biology with many patients having AD mixed with other pathology, some having pure AD, and some having non-AD phenocopy disorders [70]. How this biological heterogeneity may affect treatment response is unknown.

Despite the widespread use of antidepressants (30–50% of patient with AD/dementia are on antidepressants), there is mixed evidence regarding the benefits from their use in depression of AD patients. Individual studies have shown a trend toward tricyclic antidepressants (TCA), selective serotonin reuptake inhibitors (SSRIs), and serotonin and norepinephrine reuptake inhibitors (SNRI), being effective in management of depressive syndromes in patients with AD but meta-analyses do not support a reliable benefit [71]. Many trials have been carried out on small numbers of patient and were underpowered to detect drug-placebo differences. Variable trial methods, comorbid conditions and differences in the administered antidepressants further confound findings [72]. One SSRI trial with sertraline was positive, but three others with sertraline, fluoxetine, citalopram, and escitalopram were not [73]. While several smaller trials have found SSRIs to be effective, the Depression of Alzheimer’s Disease-2 (DIADS-2 trial) a large, multicenter trial found no difference between sertraline (100 mg) and placebo in patients with depression of AD [74–76•]. The study showed that the sertraline group experienced more side effects (diarrhea, dizziness, and dry mouth) and pulmonary serious adverse events. Treatment with sertraline was not associated with significantly greater reductions in caregiver distress than placebo [77]. Escitalopram (citalopram’s metabolite), although safe, failed to show significant antidepressant benefit for AD patients in one study [73].

In cases of severe depression when the patient is suicidal or when other NPS cluster together (e.g., agitation or/and aggression) or in situations of high risk of self- harm or self-neglect, general guidelines for treating depression in the geriatric population should be considered. The American Psychiatric Association (APA) recommends a trial of an antidepressant to treat clinically significant, persistent depressed mood in patients with dementia, and SSRIs are preferred because of their favorable safety profile [78]. Sertraline, citalopram, or escitalopram in low doses are the most appropriate first-line agents. Other SSRIs like fluoxetine and paroxetine are not recommended as a first-line SSRI due to uncertain efficacy or unfavorable (mostly anticholinergic) side effects [79]. The dose might be incrementally increased, if tolerated, to a maximum of 150 mg of sertraline or 40 mg of citalopram per day with close monitoring of side effects. Although improvement should occur within 4 to 6 weeks at the target dose, a longer period may be required to reach full effect [80].

If patients do not respond to the SSRI, switching to a different agent or augmenting treatment with a second agent should be considered. Following the “therapeutic metaphor” mentioned above, an atypical antipsychotic in a small dose is appropriate for patients who have psychotic symptoms or agitation along with depression [45, 81]. An anticonvulsant in smaller doses (the best evidence is for carbamazepine) might be considered as additional therapy to an antidepressant if there is moderate or severe agitation [56].

Switching antidepressants to a different class (as opposed to augmentation) is recommended in cases with severe side effects induced by the initial medication. Preferred second-line agents are SNRIs such as venlafaxine or duloxetine or antidepressants with a mixed pharmacology (mirtazapine, bupropion). Evidence for benefit from use of non-SSRI antidepressants specifically for depression in AD is lacking. Tricyclic antidepressants are generally not recommended due to anticholinergic side effects [82].

Psychiatric hospitalization should be considered an option in cases where self-harm is a threat. For patients with severe, refractory depression, electroconvulsive therapy (ECT) might be considered, especially if there is risk of self-harm or harm to others [80, 83].

Vortioxetine has a unique pharmacologic profile inhibiting the serotonin (5-HT) transporter and acting as a serotonin receptor agonist (5-HT1A), a partial agonist (5-HT1B), and an antagonist (5-HT3, 5-HT7, and 5-HT1D) at different receptors [84••]. This agent is of interest in NDD because in major depression, it has shown benefit on the digit symbol substitution test independent of its effect on mood and suggesting a beneficial effect on executive function. The effects have not been adequately studied in AD or other NDD.

### Treatment of Apathy in AD

Dopaminergic reward circuitry dysfunction is strongly implicated in the symptoms of apathy in AD and drugs enhancing dopaminergic transmission have been proposed as treatment. Methylphenidate acts by blocking the dopamine and norepinephrine transporters, leading to increased concentrations of synaptic dopamine and norepinephrine. This effect in turn leads to increased dopamine and norepinephrine at receptor sites. Methylphenidate is also a weak 5-hydroxytryptamine-1A (5HT1A) receptor agonist.

Methylphenidate, but not modafinil, was shown to be effective in reducing apathy in AD in a small cross-over trial [85] and was further assessed in the larger, multicenter, double blind trial—Alzheimer’s Disease Methylphenidate Trial (ADMET) [86]. In ADMET, methylphenidate (20 mg daily for 6 weeks) was associated with a significant reduction in apathy symptoms. Two study outcomes measures—the Clinical Global Impression of Change (CGI-C) and NPI apathy score—showed diminished apathy with methylphenidate treatment. A trend toward increased Mini-Mental State Examination (MMSE) scores suggested that methylphenidate treatment was associated with improved global cognition. Adverse events and side effects were modest. The results suggest that methylphenidate treatment may have clinical utility in treating apathy of AD.

### Treatment of Psychosis in PD

Pimavanserin is a selective-serotonin 5-HT2A inverse agonist. It is approved by the FDA for the treatment of hallucinations and delusions of PD psychosis. Approval of pimavanserin was based on the results of a trial in which adults with PD psychosis were randomly assigned to take 40 mg of pimavanserin or placebo daily for 6 weeks [30]. Patients taking pimavanserin experienced fewer and less severe hallucinations and delusions without worsening of the primary motor symptoms of PD. The most common adverse effects reported by patients taking pimavanserin included peripheral edema, nausea, and confusion.

Clozapine is efficacious in the treatment of PD psychosis as shown by randomized controlled trials of even very small doses (6.25 to 50 mg daily) [87, 88]. The risk of agranulocytosis and the necessity of blood monitoring with clozapine have led many experts to recommend a trial of other antipsychotics, mainly quetiapine (12.5–150 mg) before implementing use of clozapine [28, 88].

Efforts to treat psychosis of PD with antipsychotics commonly used for schizophrenia have shown limited efficacy and were associated with significant deterioration of the motor symptoms [89, 90]. Typical antipsychotics, especially potent blockers of dopaminergic receptors, are contraindicated in PD because of motor worsening.

Quetiapine is the most frequently prescribed agent targeting psychotic symptoms in PD. Trial findings are, however, inconsistent, and firm conclusions about its efficacy cannot be drawn. One open-label trial found a significant improvement in psychotic symptoms (assessed by the Brief Psychiatric Rating Scale [BPRS]) in PD patients treated with quetiapine for 12 weeks [91]. In this study, the benefit of quetiapine (mean dose 91.5 mg/daily) was comparable with a benefit of clozapine (mean dose 26 mg/daily). Several studies report significant improvement in the level of global clinical functioning of the PD patients with psychosis treated with quetiapine (Clinical Global Impression-Improvement [CGI-I] and Clinical Global Impression-Severity [CGI-S]) [91–93]. A meta-analysis of data from different trials including a total of 241 participants randomized to either quetiapine or a comparator (placebo or clozapine) [93] failed to support efficacy. All the studies show that patients taking quetiapine experienced fewer side effects than occur with other antipsychotics, but the efficacy superior to placebo was not demonstrated.

Risperidone was beneficial in managing psychotic symptoms of PDD as assessed by the BPRS and CMAI scores. In the same group, treatment with risperidone improved levels of social, occupational, and psychological functioning [94]. An unfavorable safety profile limits risperidone use.

Olanzapine was assessed for treatment of PD psychosis, but worsening of motor function and overall psychiatric symptoms were reported in up to 80% of individuals [28].

### Treatment of Depression in PD

Several studies demonstrate efficacy of antidepressants for PD depression. Ideally, pharmacological and non-pharmacological intervention should be initiated concomitantly to promote optimal response in the management of PD depression.

Dopamine agonists are commonly used as a first step in the management of depression of PD if the patient is not already receiving this treatment. Studies show pramipexole (range of doses 0.3–4.2 mg/day) and ropinirole (10 mg/day) have antidepressant properties in patients with PD [95, 96]. In a 12-week randomized double-blind, placebo-controlled trial of 323 patients, pramipexole was found to improve mood independently of changes in motor function [95]. Similarly in a longer (8-month) prospective randomized study of 41 patients, pramipexole but not pergolide was effective in reducing depression [96]. As an adjunct medication, ropinirole (daily dose of 10 mg) improved both anxiety and depressive symptoms in PD patients with motor fluctuations [97]. Despite benefits, dopamine agonist use may be limited since this group of medications is linked to risk for developing impulse control disorders. Regular monitoring to capture symptoms such as pathologic gambling, hypersexuality, and overspending in the course of dopamine agonist therapy is crucial [98].

If an antidepressant agent is warranted and there is no cognitive impairment, SSRIs or TCAs can be considered as first-line therapy. The TCAs inhibit reuptake of dopamine, norepinephrine, and, to a lesser extent, serotonin. The TCAs also possess alpha-adrenergic antagonist, antimuscarinic, and antihistaminic properties. Almost all TCAs—amitriptyline, desipramine, imipramine, and nortriptyline (with the exception of doxepine)—have been demonstrated to be potent antidepressants in randomized controlled trials involving patients with PD. The secondary amine TCAs (e.g., desipramine and nortriptyline) are preferred due to better tolerability over the tertiary TCAs. Desipramine and imipramine improved motor symptoms of PD as well as mood [28, 99]. In a subset of patients, such as those with hypersalivation or overactive bladder, the antimuscarinic activity of TCAs may be of additional clinical benefit. Sedating TCAs may be helpful for the treatment of depression with insomnia. Anticholinergic properties of TCAs limit their use in PD patients with existing or emerging cognitive dysfunction. Although infrequent, the TCAs have the potential to induce cardiac conduction disturbances, and obtaining a baseline electrocardiogram prior to treatment initiation is recommended for best practice.

If the use of a TCA is limited or contra-indicated due to cognitive impairment, comorbid medical conditions, or treatment-emergent adverse effects, SSRIs can be considered as first-line treatment. In a 30-day randomized, double-blind, placebo-controlled trial, desipramine and citalopram both improved depressive symptoms compared with placebo [100]. In practice, SSRIs are well tolerated and with the exception of tremor, which is occasionally induced by SSRIs. They rarely exacerbate PD motor symptoms. In uncontrolled studies, citalopram, escitalopram, fluoxetine, fluvoxamine, paroxetine, and sertraline were similarly effective for the treatment of depression in PD [101•]. Among the SSRIs, citalopram and sertraline are preferred due to efficacy results, tolerability, and low drug-drug interaction potential. Paroxetine is a less favorable choice due to its profile of side effects and the observations that it failed to demonstrate superiority over placebo in some randomized trials [99].

In addition to SSRIs and TCAs, several other antidepressants including bupropion, duloxetine, mirtazapine, moclobemide, nefazodone, and venlafaxine have been evaluated in controlled trials for the management of depression in PD. Both open-label and controlled data demonstrate that duloxetine and venlafaxine are well tolerated and improve depressive symptoms in patients with PD. In a 12-week open-label study of 151 patients, duloxetine was found to be well tolerated and improved depressive symptoms in patients with PD [102]. In another 12-week randomized, double-blind, placebo controlled study of 115 patients, venlafaxine-extended release was found to be as effective as paroxetine for improving depression in PD; both agents were superior to placebo [101•]. SNRIs however were found to have lower acceptability and tolerability than SSRIs in PD [102].

Non-selective monoamine oxidase (MAO) inhibitors (isocarboxazid, phenelzine, and tranylcypromine) should be avoided in levodopa-treated patients because of the risk of hypertensive crisis. The selective monoamine oxidase (MAO)-B inhibitors may be beneficial in the treatment of PD depression. An open-label, randomized, prospective, parallel group study of augmentation of levodopa with selegiline, a MAO-B inhibitor, showed that it prevented progression of minor depression in PD over a 1-year interval. MAO-B inhibitors represent an alternative treatment for patients not responsive to more widely used first choice antidepressants.

Mirtazapine, a multiple-mechanism antidepressant (presynaptic alpha-2 antagonist blocker of 5-HT2a, 5-HT2c, 5-HT3, and H-1 receptors), is a promising treatment option for depression of PD. In a randomized double-blind, placebo-controlled study involving 20 depressed PD patients, mirtazapine (30 mg/day) in combination with brief psychotherapy was superior to placebo in reducing depression [103]. Preliminary evidence suggests that mirtazapine may improve tremor and may ameliorate levodopa-induced dyskinesias [104].

ECT should be considered for patients with severe and treatment resistant PD-related depression especially if complicated by psychosis or when the patient is at high risk for self-harm. Safety of ECT is acceptable and does not produce additional cognitive impairment [105]. Motor symptoms—as well as the mood disorder—of PD often improve in the course of ECT [105].

## Agents in Clinical Trials for Treatment of Neuropsychiatric Syndromes in Neurodegenerative Disorders

Review of the available treatments for NPS in NDD demonstrates the need for better understanding of the neurobiology of NPS, more information on behavioral subtypes and their treatment responsiveness, improved interventions for patients with NPS, novel trial designs to more rapidly assess efficacy and safety, and innovative approaches to regulatory review.

Table 2 summarizes the agents currently in clinical trials for neuropsychiatric syndromes in patients with neurodegenerative diseases. Agitation in AD is a particularly active area with 11 trials currently in progress. There is substantial innovation in the mechanisms of actions of these agents and there is extensive use of repurposing as a strategy for developing new therapies. For example, gabapentin, escitalopram, carbamazepine, mirtazapine, and lithium are all repurposed agents that are approved for specific indications and are being assessed in clinical trials for agitation in patients with AD. Novel agents include multi-receptor molecules such as ITI-007 and AVP-786.

**Table 2:** Drugs in currently active double-blind placebo-controlled clinical trials for neuropsychiatric aspects of neurodegenerative disorders (from clinicaltrials.org; accessed May 21, 2018)

Disorder	Neuropsychiatric syndrome	Agent	Phase	Sponsor
Alzheimer’s disease	Agitation	Gabapentin	4	University of Texas, Austin
	Agitation	Pimavanserin	2	ACADIA Pharmaceuticals
	Agitation	Dronabinol	2	Johns Hopkins University
	Agitation	AVP-786	3	Avanir Pharmaceuticals
	Agitation	Escitalopram	3	Johns Hopkins Bloomberg School of Public Health
	Agitation	Nabilone	3	Sunnybrook Health Sciences Center
	Agitation	Carbamazepine and mirtazapine	3	University of Success
	Agitation or psychosis	MP-101	2	Mediti Pharmaceuticals Inc
	Agitation	Lithium	2	New York State Psychiatric Institute
	Agitation (mild)	Piromelatine	2	Neurim Pharmaceuticals Ltd
	Apathy	Methylphenidate	3	Johns Hopkins Bloomberg School of Public Health
	Sleep	Zolpidem; zoplicone	3	Brasilia University Hospital
		Lemborexant	2	Eisai Inc.
		Suvorexant	3	Merck Sharp & Dohme Corp.
Dementia with Lewy bodies	REM Sleep Behavior Disorder	Nelotanserin	2	Axovant Sciences Ltd.
Parkinson’s disease	Psychosis	SEP-363856	2	Sunovion
	REM Sleep Behavior Disorder	Nelotanserin	2	Axovant Sciences Ltd.
	REM Sleep Behavior Disorder	Melatonin and clonazepam	2	Seoul National University Hospital
	Excessive sleepiness	JZP-110	2	Jazz Pharmaceuticals
	Sleep disturbances	Melatonin	4	KIMJisun
	Depression (inadequately controlled)	Pimavanserin	2	ACADIA Pharmaceuticals
	Impulse control disorder	*N*-acetylcysteine	3	Center Hospitaliere Universitaire, Amiens
Huntington’s disease	Irritable mood	SRX246	1/2	Azevan Pharmaceuticals
Dementia*	Psychosis	Pimavanserin	3	ACADIA Pharmaceuticals

*Dementia-related psychosis includes psychosis occurring in Alzheimer’s disease, vascular dementia, frontotemporal dementia, progressive supranuclear palsy, and corticobasal degeneration

Sleep dysregulation in NDD is another area in which there is substantial interest. There are three sleep studies in AD. Some trials use traditional hypnotics such as zolpidem and zoplicone; others utilizing novel pharmacologic approaches are evaluating orexin antagonists including suvorexant and lemborexant. Sleep is addressed in a nelotanserin trial of REM sleep behavior disorder in patients with DLB. In addition, there is a nelotanserin study of REM sleep behavior disorder in PD; a study of melatonin and clonazepam in REM sleep behavior disorder in PD; an investigation of JZP-110 for excessive sleepiness in PD; and a study of melatonin for sleep disturbances in PD. There is a trial of SEP-363856 for the treatment of PD psychosis. Additional studies in PD include a study of pimavanserin for inadequately controlled depression and *N*-acetylcysteine for impulse control disorder in PD. There is a study of irritable mood using SRS246 in patients with Huntington’s disease.

Innovation in clinical trial design is also apparent in drug development programs. A novel approach in drug development for NPS of NDD is the trial of pimavanserin for treatment of dementia-related psychosis (DRP). DRP includes psychosis occurring in patients with AD, vascular dementia, FTD, PSP, and CBD. The structure of this study assumes that there is a final common pathway for the emergence of psychosis in patients with NDD and that modulation of this pathway with a 5-HT2A inverse agonist will result in amelioration of psychotic symptoms across the different NDD types. This trial is also innovative in having a withdrawal design with all patients placed on pimavanserin at the beginning of the trial; those who improve are eventually withdrawn in a double-blind phase of the trial. Withdrawal trials have the advantage of putting all patients onto medications when symptoms are present, responding immediately to the presence of symptoms without the threat of being placed on a placebo, and limiting the withdrawal population to those patients who responded in the treatment period. The withdrawal design limits the placebo effects that affect many trials of NPS in NDD. 

**Fig. 1:**
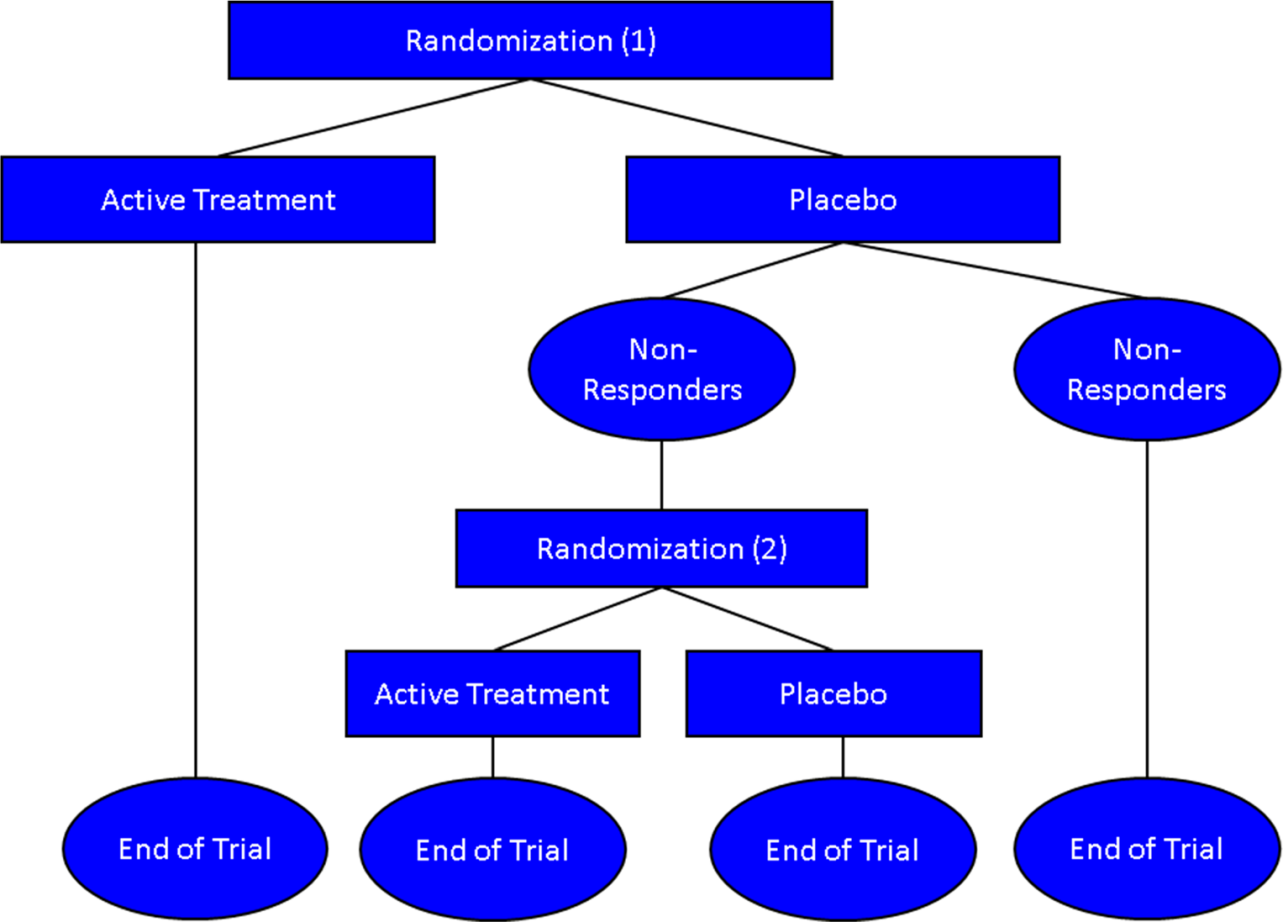
Serial parallel comparative design (SPCD). Placebo non-responders are re-randomized to drug or placebo

Another means of exploring and limiting placebo effects is the sequential parallel comparison design (SPCD) in which a 2-stage approach is used with the first consisting of a standard randomization to drug or placebo and the second consisting of a re-randomization of placebo non-responders to drug or placebo [106] (Fig. 1). The placebo response in the second stage is typically lower than in the first and allows drug activity to be determined. The first study using this design in NDD was in a trial of dextromethorphan/quinidine for agitation in AD [26]. The trial demonstrated a robust effect of dextromethorphan/quinidine on agitation in AD.

Substantial innovation is apparent in drug development for neuropsychiatric symptoms in patients with AD, and the emergence of new therapies based on a robust pipeline of new treatments in combination with novel trial designs is promising.

## Conclusions

It is increasingly recognized that NPS play a major role in NDD diagnosis. The presence of NPS is required for the diagnosis of bvFTD, and NPS have a role in syndromic diagnosis of dementia, AD, PD, VCI, PSP, and CBD. Consensus clinical criteria have emerged for psychosis, apathy, agitation, and depression in AD and for psychosis and depression in PD. Treatment of NPS in NDD is based primarily on the “therapeutic metaphor” analogy [29] with idiopathic psychiatric disorders; increasing understanding of the neurobiology of NPS in NDD may provide new insights into how best to approach therapies of these complex syndromes. A review of clinical trials shows that antipsychotics can benefit psychosis and agitation in AD and methylphenidate can reduce apathy in AD. For psychosis of PD, pimavanserin is approved by the FDA and clozapine is often effective. Quetiapine is widely used but the data supporting its efficacy are variable. Antidepressant trials in AD have shown variable efficacy in improving mood; antidepressants consistently show benefit in improving mood in depression with PD. Clinical trials of emerging agents show that drugs in the pipeline have diversified mechanisms of action. There are many repurposed agents among pipeline treatments for NPS in NDD. Innovation in clinical trial designs for NPS is apparent. The pipeline promises to deliver new and more effective therapies for NPS in NDD.
